# The clinical meaningfulness of ADAS-Cog changes in Alzheimer's disease patients treated with donepezil in an open-label trial

**DOI:** 10.1186/1471-2377-7-26

**Published:** 2007-08-30

**Authors:** Kenneth Rockwood, Sherri Fay, Mary Gorman, Daniel Carver, Janice E Graham

**Affiliations:** 1 Division of Geriatric Medicine, Department of Medicine, Dalhousie University, Halifax, Canada; 2 St. Martha's Regional Hospital, Antigonish, Canada; 3 Department of Bioethics, Faculty of Medicine, Dalhousie University, Halifax, Canada

## Abstract

**Background:**

In 6-month anti-dementia drug trials, a 4-point change in the Alzheimer's Disease Assessment Scale-Cognitive Subscale (ADAS-Cog) is held to be clinically important. We examined how this change compared with measures of clinical meaningfulness.

**Methods:**

This is a secondary analysis of a 12 month open-label study of 100 patients (71 women) diagnosed with mild to moderate AD treated with 5–10 mg of donepezil daily. We studied the observed case, 6-month change from baseline on the ADAS-Cog, the Clinician's Interview Based Impression of Change-Plus Caregiver Input (CIBIC-Plus), patient-Goal Attainment Scaling (PGAS) and clinician-GAS (CGAS).

**Results:**

At 6 months, donepezil-treated patients (n = 95) were more likely to show no change (+/- 3 points) on the ADAS-Cog (56%) than to improve (20%) or decline (24%) by 4-points. ADAS-Cog change scores were little correlated with other measures: from -0.09 for PGAS to 0.27 for the CIBIC-Plus. While patients who improved on the ADAS-Cog were less likely to decline on the clinical measures (26%), 43% of patients who declined on the ADAS-Cog improved on at least two of the clinical measures.

**Conclusion:**

The ADAS-Cog did not capture all clinically important effects. In general, ADAS-Cog improvement indicates clinical improvement, whereas many people with ADAS-Cog decline do not show clinical decline. The open-label design of this study does not allow us to know whether this is a treatment effect, which requires further investigation.

## Background

Cholinesterase inhibition is a strategy for treating Alzheimer's disease (AD) that yields statistically significant though modest cognitive benefits which favour treatment over placebo[1,2]. The clinical meaningfulness of cholinesterase inhibition remains controversial [3-7]. A widely used method of inferring clinical importance is to classify patients by whether they demonstrate a clinically meaningful minimal difference on an outcome measure [8]. The Alzheimer's Disease Assessment Scale-Cognitive subscale (ADAS-Cog) is the *de facto *standard primary outcome neuropsychological measure for AD trials [9]. It measures several cognitive domains, including memory, language and praxis. Total scores range from 0–70, with higher scores (≥ 18) indicating greater cognitive impairment. Many regulatory authorities recognize a four-point change on the ADAS-Cog at 6 months as indicating a clinically important difference, a proposal that has impacted how clinical trials are interpreted [10-13]. Our group was interested in understanding whether a four-point change on the ADAS-Cog was reflected in changes on other, more self-evidently meaningful clinical measures.

## Methods

### Sample

These data come from a previously reported 12-month, open label trial of 100 community dwelling, mild-to-moderate Alzheimer's disease patients (71% women; average age = 76 years ± 8) treated with donepezil. The Atlantic Canada Alzheimer's disease Investigation of Expectations (ACADIE) Study was conducted between 1998–1999 [14,15]. Diagnoses were made using standard criteria [16,17]. Staging followed the Clinical Dementia Rating (CDR) scale (mild = 75%) [18]. All patients were treated with 5 mg/day of donepezil for three months, and then flexibly dosed at 5 or 10 mg/day. Here, for better comparison with the 6-month double-blinded trials, we included only those patients who received treatment for a minimum of six months. To ensure that we would address only the meaningfulness of true change, we did not impute in the case of missing data.

### Outcome measures

We compared the 6-month responses on the ADAS-Cog with those from three judgment-based clinical measures. The primary outcome was Goal Attainment Scaling (GAS), used to evaluate patient-centred outcomes [19]. GAS allows clinicians and patients/caregivers to selectively target symptoms, specify desired treatment outcomes (goals), and evaluate the extent to which these goals are met. We used a modified GAS approach, setting goals on a 5-point scale anchored at 0 (baseline) [14]. The 5 points correspond to individualized descriptions of the pre-treatment state (baseline, recorded as 0), desired improvements ('somewhat' and 'much' better than baseline, recorded as +1 and +2), and potential worsening ('somewhat' and 'much' worse, recorded as -1 and -2). For example, the baseline status (level 0) for a person with a misplacing problem might be described as follows: *misplaces commonly used items, such as glasses, keys, TV remote, and wallet as often as 8 times per day and cannot locate items without verbal direction or hands-on assistance*. The goals of treatment (desired improvements) might be the ability to find misplaced items without assistance at least once per day (level +1), and misplacing items fewer than 3 times per day (+2). Goals can then be weighted or ranked in order of their relative importance (the most important goal receives the highest numerical rank). Goal attainment is evaluated by comparing the current status at follow-up with the baseline status and determining where that outcome should be slotted on the scale; attainment is recorded as 0 if there has been no change, but is otherwise scored from -2 to +2. Individual goal scores are summarized for each patient using the following formula: 50 + {(10∑(w_*i*_x_*i*_))/(0.7(∑w_*i*_)^2^)^1/2^}, where w_*i *_= weight assigned to the *i*th goal and x_*i *_= score of the *i*th goal). The summary score is 50 when all goals remain at the baseline level, greater than 50 when there is more improvement across goals than decline, and less than 50 with worsening. In ACADIE, treatment goals were constructed separately by clinicians (CGAS) and patients/caregivers (PGAS). Each was blinded to the goals set by the other. Only patient/caregiver goals were weighted (for each CGAS goal, weight = 1). Goals were coded into five domain categories: cognition, function, behaviour, leisure and social interaction. Examples of the types of goals that were set for each domain include: cognition – a decrease in repetitive questioning, improved word finding, improvement in recent memory, less misplacing of objects; function – performing various IADL and ADL tasks with less dependence; behaviour – less irritability, more initiative; social activities – outings, especially to scheduled activities such as church, bingo, card games; leisure – more interest in or effective performance of hobbies and pastime activities. The ADAS-Cog was completed independently of the CGAS, and although patients might have had some idea as to how they performed on the ADAS-Cog, neither they nor their caregivers were told of the ADAS-Cog scores.

The Clinician's Interview Based Impression of Change – Plus Caregiver Input (CIBIC-Plus) was a secondary outcome [20]. This global assessment of change is based on a comprehensive, semi-structured, patient/caregiver interview, anchored at 4 ('no change') and ranges from 1–7, where 1 is "very much improved" and 7 is "very much worse." Other secondary outcomes included the Mini-Mental State Examination (MMSE) scored between 0–30 (lower scores indicate greater cognitive decline), the Lawton-Brody Physical Self-Maintenance (PSMS) and Instrumental Activities of Daily Living (IADL) scales, which range from 6–30 and 6–31, respectively (higher score indicate less functional ability), and the Cornell Scale for Depression in Dementia, with a range of 0–38 (scores greater than 6 indicate depression) [21-24]. The latter three measures rely on informants. All measures were administered at baseline, then every three months up to 12 months.

### Analyses

In this exploratory study, we analyzed observed cases (OC) after 6 months of donepezil therapy. ADAS-Cog change scores ≤ -4 were equated with improvement, whereas worsening was defined as a change score ≥ 4. Scores between 3 and -3 were interpreted as maintenance of the baseline status (no change). We report the frequency, proportion and baseline characteristics of patients in each ADAS-Cog response group. Mean change from baseline, standard deviation and 95% confidence intervals were calculated for all outcome measures by ADAS-Cog response group. Between group differences were tested using chi-square. Spearman correlation coefficients were calculated to compare mean change on the ADAS-Cog, as well as the CIBIC-Plus and GAS, with response on the other outcome measures. Statistical tests were interpreted at the 5% significance level.

Cut-points were set for each of the judgment based measures to reflect clinically detectable changes. CIBIC-Plus improvement was taken as scores of 1–3, and worsening as 5–7. GAS responses were grouped so that PGAS improvement was defined as a change > 6 (representing net improvement in 2/3 of the 8.6 goal areas set on average by patients and caregivers, and a standardized response mean of moderate size ~ 0.6), worsening as a change < -6, and no change as scores between 5 and -5. Respective CGAS cut-points were set as > 3, < -3 and 2 to -2, again representing net improvement on most goals, as clinicians set 3.4 goals on average, and also a standardized response mean in the moderate range of clinical detectability. In this way, responses on each of the three judgment-based clinical measures could be cross-classified against the ADAS-Cog.

### Ethics

All patients and caregivers provided written informed consent. The study protocol was approved by the Research Ethics Committee of the Queen Elizabeth II Health Sciences Centre, Halifax, Nova Scotia, any by the institutional ethics boards at each participating study centre.

## Results

### Demographics and baseline characteristics

Ninety-five of 100 patients enrolled at baseline were evaluated at 6 months. Five patients had discontinued; three due to adverse events (diarrhoea n = 2, weight loss n = 1) and two withdrew consent. The remaining patients tended to be elderly women with mild AD (Table 1), the majority of whom (63%) were dosed at 10 mg donepezil after the initial 3-month follow-up.

**Table 1:** Baseline characteristics of patients with mild-moderate Alzheimer's disease who were treated with donepezil for 6 months, by ADAS-Cog response group

	*All patients *	*ADAS-Cog*	*ADAS-Cog *	*ADAS-Cog*
	*(n = 95)*	*improved *	*no change*	*worsened *
		*(n = 19)*	*(n = 53)*	*(n = 23)*
**Baseline characteristics**				
Females, n (%)	68 (72)	15 (79)	36 (68)	17 (74)
Age, mean years (sd)	75.8 (7.7)	79.8 (8.2)	75.5 (6.3)	73.3 (8.9)
Education, mean years (sd)	11.1 (3.2)	10.5 (3.2)	11.0 (3.2)	11.6 (3.2)
Duration of illness, mean years (sd)	1.3 (1.5)	1.0 (1.0)	1.3 (1.7)	1.5 (1.6)
CDR = mild, n (%)	73 (77)	14 (74)	44 (83)	15 (65)
**Baseline outcomes**				
CIBIS+, mean (sd)	3.7 (0.8)	3.7 (0.8)	3.6 (0.7)	3.9 (0.7)
MMSE, mean (sd)	19.9 (5.3)	20.0 (4.7)	20.6 (5.3)	18.3 (5.4)
ADAS-Cog, mean (sd)	24.6 (9.9)	26.4 (10.6)	23.2 (9.3)	26.3 (10.4)
PSMS, mean (sd)	8.8 (2.5)	9.3 (2.1)	8.8 (2.7)	8.3 (2.2)
IADL, mean (sd)	19.9 (5.4)	20.5 (4.6)	20.1 (5.4)	18.9 (6.1)
CSD, mean (sd)	5.7 (5.4)	6.4 (5.5)	5.5 (5.1)	5.9 (6.2)
**Treatment goals set at baseline**				
PGAS-total, mean (sd)	8.6 (3.3)	8.2 (2.7)	8.7 (3.3)	8.6 (3.8)
PGAS-cognition, mean (sd)^1^	1.2 (0.8)	1.1 (0.7)	1.2 (0.8)	1.2 (0.7)
CGAS-total, mean (sd)	3.4 (1.3)	3.1 (0.8)	3.5 (1.4)	3.3 (1.2)
CGAS-cognition, mean (sd)^2^	2.1 (1.8)	2.5 (2.3)	2.2 (1.7)	1.8 (1.4)

^1 ^81/95 patients/caregivers set cognition goals: respectively 16/19, 45/53 and, 20/23

^2 ^81/95 clinicians set cognition goals, respectively 17/19, 45/53 and 19/23

### ADAS-Cog response at 6 months

The most common response on the ADAS-Cog at 6 months was no change from baseline (56%, mean change = -0.1, ± 2.0). Patients who showed worsening (24%; mean change = 8.0, ± 4.7) outnumbered those who had improved (20%; -6.2, ± 1.7) Patients who improved on the ADAS-Cog were slightly older than those in the other response groups, but there was no clear effect of initial conditions – *i.e*. those who responded showed no statistically significant differences from non-responders in baseline clinical or demographic measures (Table 1).

### Comparison of ADAS-Cog response with other outcomes at 6 months

**Figure 1:**
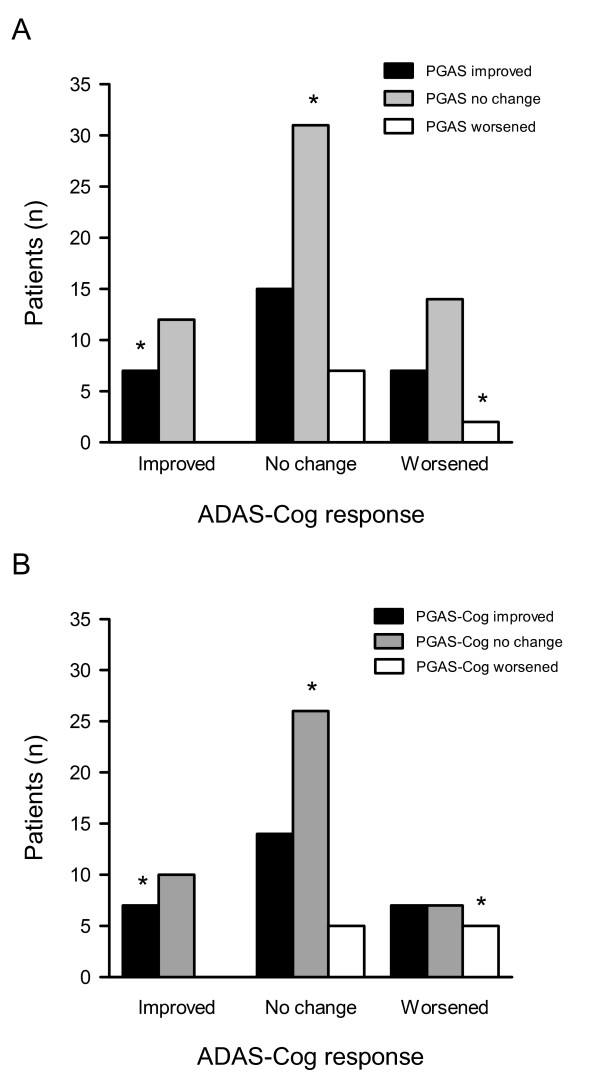
**Distribution of patients by ADAS-Cog and patient/caregiver-GAS response after 6 months of donepezil therapy**. Patient responses on the ADAS-Cog at 6 months were categorized as improved (change from baseline ≤ -4 points), no change (-3 to 3) or worsened (≥ 4) and crossed against patient/caregiver-GAS (PGAS) improvement (change from baseline ≥ 6), no change (-5 to 5) or worsening (≤ -5) for total PGAS goals (A) and PGAS cognition goals (B). An asterisk (*) denotes agreement between measures – overall 42% for total PGAS and 47% for PGAS-cognition.

The most common response on the patient/caregiver-GAS (PGAS) at 6 months was no change (*i.e*., within the range -3 to + 3) which was twice as common as improvement (60% versus 31%; Figure 1, Panel A). Overall, the PGAS response did not correlate well with the ADAS-Cog response (Table 2). Only 42% of patients, mostly in the no change/no change group (33%), were similarly classified by the ADAS-Cog and the PGAS (Figure 1, Panel A). At a group level, patients with ADAS-Cog improvement had net improvement on the PGAS (mean change = 7.0 ± 9.1) compared with patients who had ADAS-Cog worsening (5.4 ± 11.2). At the individual level, however, there were differences in classification: while no one who was classified as improved on the ADAS-Cog was rated as having worsened clinically, 7/23 people who worsened on the ADAS-Cog were rated by patient/caregivers as having improved. This appears to reflect not just the broader range of domains considered in the PGAS, but also differing accounts of treatment. For example, considering only the PGAS-cognition goals (n = 81 patients), a similar pattern obtains (Figure 1, Panel B).

**Table 2:** Spearman correlation coefficients comparing mean changes on the ADAS-Cog, CIBIC-Plus, PGAS and CGAS with mean changes on other outcomes at 6 months

	*ADAS-Cog *	*ADAS-Cog *	*ADAS-Cog*				
	*improved*	*no change *	*worsened *	*ADAS-Cog*	*CIBIC-Plus *	PGAS*	CGAS*
	*(n = 19)*	*(n = 53)*	* (n = 23)*	*(n = 95)*	*(n = 95)*	*(n = 95)*	*(n = 95)*
CIBIC-Plus	-0.15	0.15	0.38	0.27	1.00		
PGAS*	0.40	-0.32	-0.15	-0.09	-0.59	1.00	
Cognition*	0.58	-0.50	-0.32	-0.22	-0.60	0.81	0.58
Function*	0.23	-0.25	-0.07	-0.03	-0.27	0.69	0.22
Behaviour*	-0.33	0.17	-0.17	-0.06	-0.12	0.47	0.18
Leisure*	0.44	-0.32	-0.15	-0.11	-0.50	0.87	0.36
Social*	0.73	-0.32	-0.50	-0.04	-0.52	0.72	0.39
CGAS*	0.33	-0.09	-0.32	-0.22	-0.77	0.52	1.00
Cognition*	0.41	-0.12	-0.27	-0.21	-0.69	0.51	0.84
Function*	0.36	-0.29	0.20	-0.14	-0.60	0.51	0.77
Behaviour*	0.20	0.35	-0.43	-0.30	-0.72	0.41	0.89
Leisure*	-1.00	0.11	0.29	0.12	-0.25	0.32	0.63
Social*	0.15	-0.31	0.21	0.01	-0.76	0.48	0.86
MMSE*	0.59	-0.18	-0.24	-0.35	-0.29	0.24	0.32
PSMS	-0.28	0.00	0.37	0.21	0.51	-0.36	-0.53
IADL	-0.29	0.16	-0.15	0.11	0.37	-0.27	-0.46
CSD	0.24	-0.03	0.39	0.25	0.40	-0.15	-0.53

*Higher scores indicate better performance (the expected direction is positive), otherwise lower scores indicate improvement.

**Figure 2:**
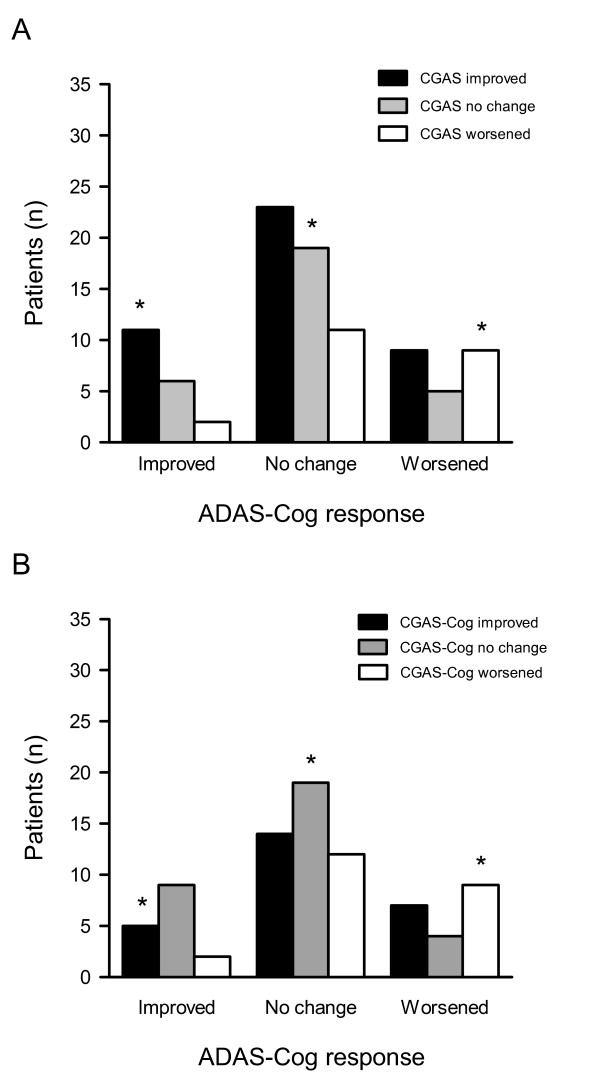
**Distribution of patients by ADAS-Cog and clinician-GAS response after 6 months of donepezil therapy**. Patient responses on the ADAS-Cog at 6 months were categorized as improved (change from baseline ≤ -4 points), no change (-3 to 3) or worsened (≥ 4) and crossed against clinician-GAS (CGAS) improvement (change from baseline ≥ 3), no change (-2 to 2) or worsening (≤ -3) for total CGAS goals (A) and CGAS cognition goals (B). An asterisk (*) denotes agreement between measures – overall 41% for both measures.

At 6-months, overall responses on the clinic-GAS (CGAS) tended towards improvement (45%), followed by no change (32%) and worsening (23%). Mean change from baseline on the CGAS corresponded with the ADAS-Cog response by group (from 5.5 ± 9.1 for ADAS-Cog improved to 0.9 ± 12.4 for ADAS-Cog worsening), but the correlation between measures was low (Table 2). Here too, agreement (41%) was concentrated primarily in the no change/no change group (20%; Figure 2, Panel A). Patients who improved on the ADAS-Cog were also more likely to have CGAS improvement (11/19), and again – as with the PGAS – less agreement was evident with ADAS-Cog worsening. Of the 23 patients worsened by ≥ 4 points, clinicians rated 9 as improved and 5 as showing no change. A similar pattern to the PGAS was also seen when we considered only cognition goals. ADAS-Cog improvement usually indicates clinical ratings of improvement or no change; whereas ADAS-Cog worsening can be seen in many people rated as showing clinical improvement (Figure 2, Panel B).

**Figure 3:**
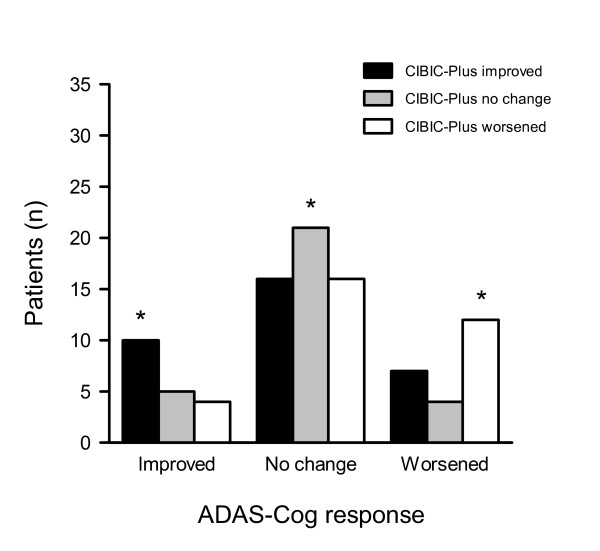
**Distribution of patients by ADAS-Cog and CIBIC-Plus response after 6 months of donepezil therapy**. Patient responses on the ADAS-Cog at 6 months were categorized as improved (change from baseline ≤ -4 points), no change (-3 to 3) or worsened (≥ 4) and crossed against CIBIC-Plus improvement (ratings of 1 to 3), no change (4) or worsening (5 to 7). An asterisk (*) denotes agreement between measures – overall 45%.

In contrast with the ADAS-Cog, the CIBIC-Plus account of change at 6 months was more evenly distributed: 35% improved, 31% had no change and 34% worsened (see Figure 3). For each ADAS-Cog response group, the mean CIBIC-Plus score changed in the corresponding direction (from 3.6 ± 1.0 for ADAS-Cog improved to 4.2 ± 1.3 for ADAS-Cog worsening), but the correlation between the CIBIC-Plus and the ADAS-Cog change scores was low-moderate (Table 2). Concordance between the CIBIC-Plus and the ADAS-Cog (*i.e*., improved on both, no change on both or worsening on both), occurred in 45% of patients, mostly in the 22% with no change by either measure (Figure 3). Clinical impressions showed less variability when the ADAS-Cog indicated improvement than for any other response. By contrast, 7/23 patients with ADAS-Cog deterioration had CIBIC-Plus scores that showed improvement, including 4 who were rated as "much" improved (CIBIC-Plus = 2). Most patients (32/53, 60%) characterized as "no change" on the ADAS-Cog were either improved (n = 16) or worsened (n = 16) on the CIBIC-Plus.

A small group of patients (15%) demonstrated consistency of response across all measure at 6 months: 5 patients who improved on the ADAS-Cog also improved on the clinical measures, 8 patients were consistent "no changers," and 1 patient worsened on all four measures. It is also notable that when the ADAS-Cog indicated improvement, only one patient worsened on more than one clinical measure, whereas 10 patients with ADAS-Cog worsening showed improvement on at least two of the three clinical measures (including 6 patients who improved on all of the clinical measures).

At 6 months, the ADAS-Cog correlated better with the MMSE than with any other outcome measure (Table 2). Here, the CIBIC-Plus correlated better than the ADAS-Cog with all other outcome measures, including PGAS and CGAS.

## Discussion

In this secondary analysis, we investigated the clinical meaningfulness of a 4-point change on the ADAS-Cog at 6 months. Patients who improved on the ADAS-Cog (n = 19) were unlikely to show clinical deterioration (none by PGAS, 2 by CGAS, and 4 by CIBIC-Plus), patients with ADAS-Cog deterioration (n = 23) have a broader range of clinical outcomes, including about a third (9 by CGAS, 7 by PGAS and 7 by CIBIC-Plus) who show clinically important improvement. In consequence, it appears that while such a 4-point ADAS-Cog change at 6 months might help regulators discriminate treatment effects between patient groups, a 4-point decline has little inherent clinical meaning for individual patient or physician decision-making. By contrast, a 4-point improvement more often signals agreement with physician and patient assessment of either improvement, or the absence of decline.

Although the "4-point change at 6 months" criterion is conventional, a recent systematic analysis of double-blind placebo-controlled trials of cholinesterase inhibitors demonstrated an average -2.7 point improvement at 6 months and one year [25]. We therefore repeated our analyses using a "3-point change" criterion at 6 months. Compared with the 4-point change criterion, this identified more people as having either improved (29 versus 19) or worsened (30 versus 23). Still, the essential point – that ADAS-Cog improvement is associated with clinical improvement whereas many people who decline on the ADAS-Cog are judged by patients and physicians to have improved- holds. The data from our study suggests that an *n*-point decline on ADAS-Cog needs to be interpreted in the context of overall response, and should not be privileged over, amongst other considerations, the preferences of patients and caregivers.

Our data must be interpreted with caution. As this is an open-label study, we cannot make any inference about whether these changes are due to treatment, and we have made no attempt to do so. Such an inference requires placebo-controlled studies. Where these data can help is in better understanding whether the commonly-accepted-as-meaningful 4-point change on the ADAS-Cog has a strong evidence base. These data make clear that changes at the group level are not easily translated into changes at the individual level. This does not mean that the ADAS-Cog account is right and that the clinical accounts are wrong, or vice versa. Neither does it gainsay that the ADAS-Cog shows, at a group level, across trials and across compounds, a dose-response effect which favours the use of cholinesterase inhibitors, compared with placebo, in people with Alzheimer's disease [2]. Nor does it endorse the view that these effects are meaningless [5]. What it appears to tell us is that we do need to look carefully at the whole body of evidence. In short, just as the CIBIC-Plus is a better estimate of decline than of improvement [26], so might the ADAS-Cog help us estimate the extent of improvement, but be less good at measuring clinically meaningful decline.

Another feature of these data is the large proportion of patients classified as 'no change' by both the ADAS-Cog and at least one other clinical measure. No change in a neurodegenerative illness can be a useful goal, but this needs to be better understood. 'No change' in the CIBIC-Plus, for example, often appears to reflect clinical trade-offs [27,28]. Whether there are detectable signals within the patients classified as no change requires additional study.

## Conclusion

The development of staging tools for untreated Alzheimer's disease, based on natural history observations, was a labour-intensive process, and took place by carefully characterizing many patients, often at single sites, over several years. To attempt the same in the changing environment of treatments for Alzheimer's disease is daunting and may well be impracticable. In the setting of ongoing clinical studies, however, especially those employing tests such as the CIBIC-Plus, there is an opportunity to systematically record clinical observations in a way that can quickly allow for some hundreds to be assembled and compared. If we cannot rely on the ADAS-Cog as a guide to individual decision-making, we need to pursue other methods, such as symptom inventories [6,29], to find more relevant and less arbitrary ways of understanding treatment effects in individual patients.

## Abbreviations

ACADIE, Atlantic Canada Alzheimer Disease Investigation of Expectations; AD, Alzheimer's disease; ADAS-Cog, Alzheimer's Disease Assessment Scale – Cognitive subscale; CDR, Clinical Dementia Rating; CGAS, Clinician's Goal Attainment Scaling; CIBIC-Plus, Clinician's Interview-Based Impression of Change, Plus caregiver input; CIBIS-Plus, Clinician's Interview-Based Impression of Severity, Plus caregiver input; CSD, Cornell Scale for Depression in Dementia; GAS, Goal Attainment Scaling; IADL, Instrumental Activities of Daily Living Scale; MMSE, Mini-Mental State Examination; PGAS, Patient/caregiver Goal Attainment Scaling; PSMS, Physical Self Maintenance Scale.

## Competing interests

Kenneth Rockwood has received honoraria and research grants from Pfizer (the study's sponsor) and its competitors Janssen-Ortho, Novartis, Lundbeck, and Merck Frosst Canada. He is part owner of DementiaGuide Inc., which runs a website to facilitate better therapeutic goal setting in dementia. Mary Gorman and Daniel Carver each have participated in CMEs sponsored by Pfizer and Janssen-Ortho. Sherri Fay and Janice Graham declare that they have no competing interests.

## Authors' contributions

KR and JG designed the ACADIE study and wrote the grant application and clinical trial protocol. KR also planned and supervised these secondary analyses and drafted the original manuscript. JG contributed to the drafting and critical revision of the manuscript. SF was Project Coordinator, assisted in the analyses and contributed to interim drafts. MG and DC were co-investigators who participated in the conduct of the study and contributed to interim drafts of this manuscript. All authors read and approved the final manuscript.

## Pre-publication history

The pre-publication history for this paper can be accessed here:


